# Hidden heterogeneity: Uncovering patterns of adherence in microbicide trials for HIV prevention

**DOI:** 10.1371/journal.pone.0267011

**Published:** 2022-05-12

**Authors:** Lori Miller, David Prieto Merino, Kathy Baisley, Richard Hayes

**Affiliations:** 1 London School of Hygiene and Tropical Medicine, London, United Kingdom; 2 Applied Statistical Methods in Medical Research Group, Catholic University of Murcia (UCAM), Murcia, Spain; IAVI, UNITED STATES

## Abstract

**Background:**

Interpretation of clinical trial results testing vaginal microbicide gels for HIV prevention depends on participant adherence. Prior to the era of antiretrovirals, microbicide trials collected adherence data via self-report, and trials typically reported trial population adherence as overall averages in primary results manuscripts. This study first sought to determine if different patterns of adherence from three trials of vaginal microbicide gels could be identified, using self-reported data and if so, how those patterns compare across trials. The second objective was to explore which individual-level factors were associated with different adherence patterns.

**Methods:**

Data from the following three clinical trials of vaginal microbicides were used for this study: HIV Prevention Trials Network (HPTN) 035 testing PRO 2000 and Buffergel, the Microbicides Development Programme (MDP) 301 testing PRO 2000, and the Population Council’s Carraguard study, testing Carraguard gel. Latent Class Analysis (LCA) was used to identify longitudinal patterns of adherence using self-reported data about gel use. Multinomial multivariable logistic regression was used to estimate relative risk-ratios for factors which were independently associated with different latent adherence trajectories within each trial, and compared across trials.

**Results:**

Included in this analysis are 2,282 women from HPTN 035 (age 17–56 years), 6238 women from MDP 301 (age 16–75 years), and 6039 women from Carraguard (age 16–73 years). Using LCA, 3–4 different patterns of gel adherence were identified in each trial; these patterns were similar across the trials. Factors associated with adherence patterns were identified in all trials. Older age was associated with the adherence trajectory that consistently reported gel use in three trials. Participant-reported negative reaction of partners to the gel was associated with trajectories that reported less consistent adherence in two trials. A greater number of baseline-reported sex partners or sex acts was associated with trajectories which reported less consistent adherence in some trials. Trial site location was associated with membership of trajectories in all trials.

**Conclusion:**

LCA was able to identify patterns of microbicide gel adherence in clinical trials that used self-reported data. Key factors associated with patterns of adherence in this study were participant age, clinical trial site location, and partner reaction to the study gel. These findings, in particular, age and perceived partner reaction to the method, are consistent with results from other clinical trials and programmatic rollout of biomedical HIV prevention methods for women in Africa. This study contributes to the body of evidence that women need more support to navigate power dynamics within their relationships with men so that they can successfully use HIV prevention methods.

## Introduction

Understanding participant adherence to study product is critical to interpretation of results from clinical trials testing vaginal gel microbicides to prevent sexual transmission of HIV. As vaginal gel microbicides are user-controlled products, women may or may not use these topically-applied investigational products according to instructions. Therefore, a trial result that does not show a reduction in HIV incidence in the active gel arm might be due to an investigational product not having sufficient biological efficacy, or it might be due to low usage by trial participants. This is particularly a problem because trial participants often report good adherence whether or not they have used the gels consistently. The challenges of adherence–both of low adherence in trials [1–3] and, for trials relying on self-reported data [4–12], not knowing the cause of null results–have hampered the microbicide field over time [13]. While these challenges have motivated funders such as the National Institute of Allergy and Infectious Diseases to re-focus efforts on methods of HIV prevention such as vaginal rings and long-acting pre-exposure prophylaxis (PrEP) [14], women need a range of options [14–16]. Community stakeholders and researchers support continued development of on-demand products [14, 17].

While adherence is a key factor in understanding trial results, primary-results publications typically provide self-reported adherence to microbicides as overall averages for trial populations. These averages can create an impression of overall high adherence to the study product. In reality, each woman has her own trajectory of adherence during her follow-up, and a longitudinal approach to data analysis may provide a better understanding of how participants use study products over time. It would be helpful to understand if there are typical patterns of adherence that exist among trial participants, rather than adherence being characterised as static. The first objective of this study was to determine if different patterns of adherence using self-reported data from three trials of the effectiveness of coitally-dependent microbicide gels could be identified, and if so, how those patterns compare across trials. If patterns were identified, the second objective was to explore which individual-level factors were associated with different adherence patterns.

## Methods

Data from three completed non-antiretroviral vaginal-gel microbicide trials were included in this study: HIV Prevention Trials Network (HPTN) study of PRO2000 and BufferGel (HPTN035 [11]), Microbicides Development Programme’s trial of PRO2000 (MDP301 [12]), and Population Council’s trial of Carraguard (Carraguard [1]). Data for this type of analysis were not available from anti-retroviral (ARV) based microbicide clinical trials at the time this study commenced.

HPTN035 was a four-arm phase II/IIb randomised placebo-controlled trial testing 0.5% PRO 2000 gel and BufferGel for the prevention of sexual transmission of HIV against a placebo gel and a condom-only arm. A total of 3101 women were enrolled in eight sites in Malawi, South Africa, Zambia, Zimbabwe, and the US. Results indicated no preventive effect for BufferGel and a non-significant 30% reduction in HIV incidence for 0.5% PRO 2000.

MDP 301 was a three-arm phase III randomised, double-blind placebo-controlled trial testing 0.5% and 2% PRO2000 for the prevention of sexual transmission of HIV against a placebo gel. In total, 9385 women were enrolled in six sites in South Africa, Tanzania, Uganda, and Zambia. The 2% PRO 2000 arm was closed early due to futility. Trial results for 0.5% PRO2000 did not indicate an effect on HIV incidence.

Carraguard was a phase III randomised, placebo-controlled, double-blind trial to test the effect of Carraguard gel for the prevention of sexual transmission of HIV against a placebo gel. It enrolled 6202 women in three sites in South Africa. Carraguard was not shown to reduce HIV incidence in this trial.

All three trials tested vaginal-gels used just before sex. Self-reported “gel use at last sex act” was collected at monthly or quarterly visits over follow-up. Table 1 provides information about each trial including investigational product, trial population size, age range of participants, locations, type and frequency of self-reported adherence data collected (Carraguard trial used a dye stain assay as its primary adherence measure), and trial dates.

**Table 1:** Clinical trials included in the latent class analysis.

Organization name	Average adherence overall by self-report	Total number of participants	Age range of participants	Type of data	Frequency of adherence data collection	Trial dates	Locations	Planned follow-up	Actual follow-up and notes
Candidate product									
“Trial name”									
HIV Prevention Trials Network (HPTN)	81%	3,101 (This analysis: 2,282)		Categorical: Gel use at last sex act (yes/no)	Quarterly	February 2005—September 2008	Malawi, South Africa, Zambia, Zimbabwe, USA	12–30 months	Average follow-up was 20.4 months; trial closed as planned
BufferGel			17–56 years						
PRO 2000									
“HPTN 035”									
Microbicides Development Programme (MDP)	89%	9,385 (This analysis: 6,238) (includes 0.5% gel arm + placebo to 52 weeks)		Categorical: Gel use at last sex act (yes/no)	Monthly	October 2005—September 2009 (2% arm dropped February 2008)	South Africa, Tanzania, Uganda, Zambia	12 months for primary analysis	For 0.5% PRO 2000 and placebo gel arms, follow-up was 12 months as planned
PRO 2000			16–75 years						
“MDP 301”									
Population Council	96%	6,202 (This analysis: 6,038)		Categorical: Gel use at last sex act (yes/no)	Quarterly	March 2004—March 2007	South Africa	9–24 months	Trial closed as planned
Carraguard			16–73 years						
“Carraguard”									

### Identifying patterns of adherence: Latent class analysis

Latent class analysis [18] (LCA) was used to identify longitudinal patterns of adherence using self-reported data, referred to here as “latent adherence trajectories”. Answers to the question “did you use the gel at your last sex act” asked monthly or quarterly, over time, were the basis for this analysis.

LCA is a type of finite mixture modelling, which is a family of modelling techniques that allow subgroups within a larger population to be identified. The goal of LCA is to take a seemingly homogeneous population and “un-mix” it so that existing constituent subgroups are identified. The latent subgroups are identified and defined by their similar response patterns to a particular set of questions or items. Because the answers to sets of questions are directly observable, it is their particular combination of response patterns that is then able to characterise the latent subgroup. Models with different numbers of trajectories are fitted, and an optimal model with a chosen number of trajectories is selected after assessing various criteria. There is no accepted best method for model selection, thus different investigators might come to different conclusions based on their evaluations of criteria and usefulness of the various models.

The selected LCA model will have a certain number of trajectories, and two parameters are estimated. The first parameter estimates the probability of a particular response to each item at each timepoint conditional on belonging to that particular latent trajectory (in the present study the estimate is for gel use at last sex act). The second parameter estimates the proportion of individuals in the population estimated to be in each latent trajectory.

Finally, the model provides estimates for each individual of their probability of belonging to each of the latent trajectories specified in the model, based on how the individual’s observed response pattern compares to the item response probabilities estimated by the model; called the posterior probability.

For this analysis, the LCA Stata plugin [19] was used in Stata SE13 for LCA. All data–for individuals with both complete and incomplete data–were used in the analysis to model the latent trajectories, as the software uses a Full Information Maximum Likelihood [18], which assumes data are missing completely at random or missing at random.

In this analysis, models with 1–6 latent adherence trajectories were fitted for each of the three trials separately. To select the model considered to best describe adherence patterns for each trial, the following criteria were examined: trajectory shape, proportion of population belonging to each trajectory, trajectory separation, homogeneity, relative fit indices (Bayesian, adjusted Bayesian, Akaike information criteria), relative entropy, parsimony, interpretability, and model usefulness. As the objective of this analysis was to reduce a large volume of data to better understand adherence patterns, key aspects of model selection were parsimony and interpretability of the latent trajectory structures. More details about latent trajectory information for each trial and choice of model are provided in the S1 Appendix.

### Identifying factors associated with adherence patterns: Multinomial logistic regression

In this exploratory analysis examining if individual level factors might be associated with different adherence trajectories, multivariable multinomial logistic regression (Stata SE13) was used to estimate relative-risk-ratios (RRRs) and their 95% confidence intervals (CIs) for factors which were independently associated with different latent adherence trajectories within each trial. The latent adherence trajectories, derived from the LCA analysis, were based on participant answers to the question “did you use the gel at your last sex act” asked monthly or quarterly, over time. The associations between types of latent adherence trajectories and explanatory factors were then compared across trials. Latent adherence trajectory was the outcome variable, with regressions weighted by each trial participant’s posterior probability of belonging to each of the latent trajectories, to account for uncertainty in class membership in the model.

Three types of individual-level factors were considered in this analysis: demographic characteristics (site, age, education), self-reported baseline behavioural characteristics (recent number of sex partners, recent number of sex acts, condom use, anal sex, transactional sex), and study exit acceptability characteristics (variables reflecting self-reported views of the microbicide gel and participants’ reports of how they thought their partners viewed the gel). The RRRs represent the ratio of the probability of being in a particular adherence trajectory versus the reference trajectory, for one categorical group versus a reference categorical group.

For each trial, the age-adjusted association of each covariate of interest and the latent adherence trajectory was examined. Variables that showed evidence of an age-adjusted association with the latent adherence trajectory based on a likelihood ratio chi-square test of p≤0.05 were added to the multivariable multinomial logistic regression (the model) one at a time, starting with those that had the strongest evidence of an association. Each variable was added into the model and assessed using the likelihood ratio chi-square test statistic of the full model versus the model without the variable until a final set of variables were identified which had strong evidence of an association (p≤0.05) with the latent adherence trajectory membership.

### Ethical approval

This study was approved by the London School of Hygiene and Tropical Medicine (LSHTM) Ethics Committee, reference 6491. Ethical approvals for included trials are reported elsewhere [1, 11, 12]. Datasets received were fully anonymised.

## Results

### Participants

In this analysis 2,282 women from HPTN 035 were included, ranging in age from 17–56 years. 1437 women had some secondary education or higher; 844 women had a primary education or below.

6238 women were included in this analysis from MDP 301, ranging in age from 16–75 years. 1508 women had some secondary education or higher; 4730 women had a primary education or below. 6039 women were included in this analysis from Carraguard, ranging in age from 16–73 years. 1142 women had some secondary education or higher; 455 women had a primary education or below.

### Latent class model selection and patterns of adherence

**Fig 1:**
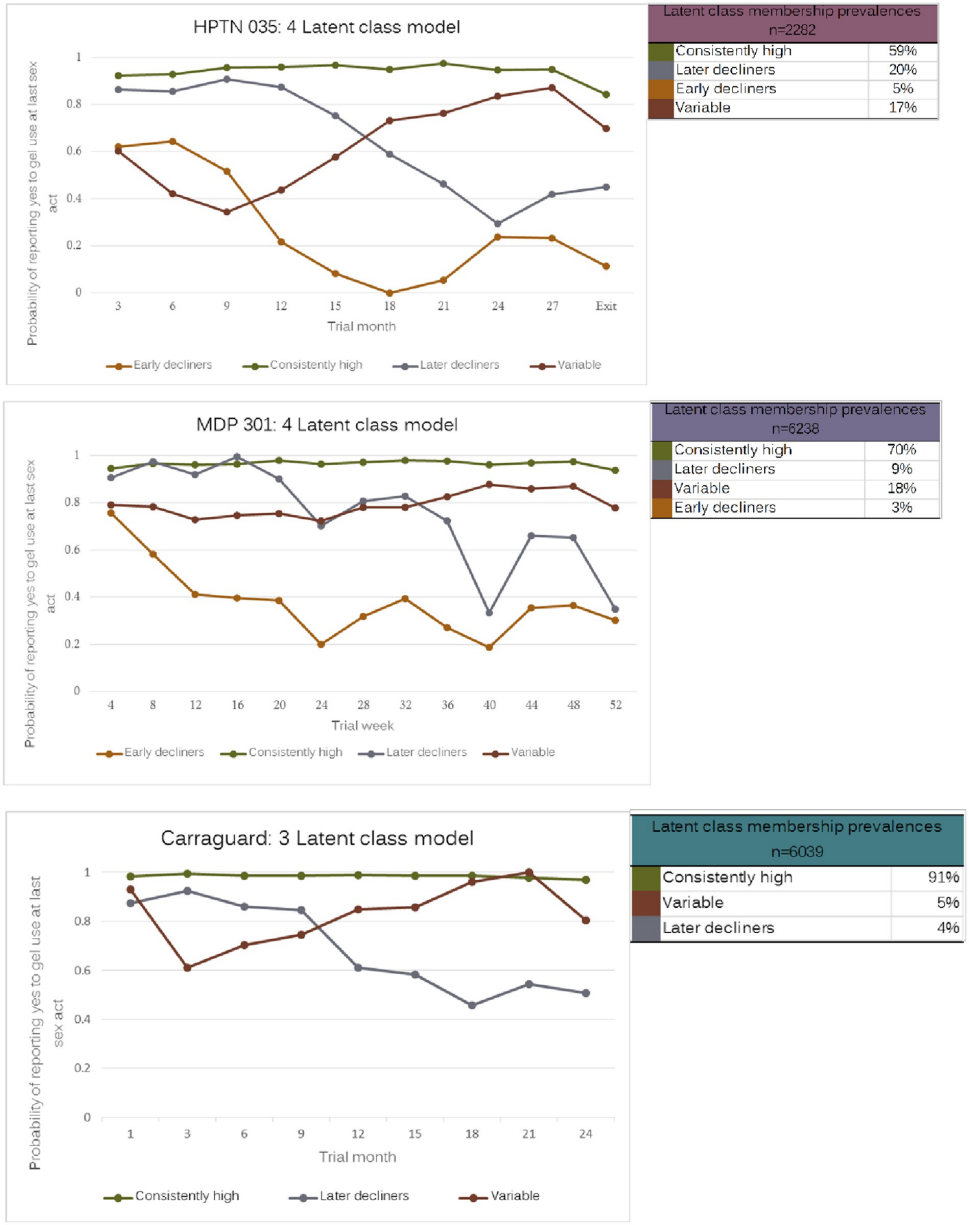
Latent class analysis models for 3 trials. Percentages may not total to 100 due to rounding error.

A model with four adherence trajectories was chosen for HPTN035 and MDP301. A model with three adherence trajectories was chosen for Carraguard. For each trial, Fig 1 shows the pattern of each adherence trajectory, and the estimated proportion of trial participants in each trajectory.

For all three trials, the largest subgroup within the trial population was the subgroup that consistently had a high probability of reporting gel use at last sex act throughout trial follow-up (“Consistently-high” subgroup: 59% HPTN035, 70% MDP301, 91% Carraguard). In all three trials, this was also the subgroup that had the least variability in its gel use reporting. In all three trials, a subgroup was identified that initially had a high probability of reporting gel use, but about midway through the trial, probability of reporting gel use at last sex act decreased and remained low for the rest of the trial (“Later-decliners” subgroup: 20% HPTN035, 20% MDP301, 5% Carraguard). A third subgroup common to all trials had variable reporting of gel use with a somewhat up-and-down pattern, initially having a high probability of reporting gel use, then a lower probability of reporting gel use, followed by an increased probability of reporting gel use. (“Variable-adherers” subgroup: 17% HPTN035, 18% MDP301, 4% Carraguard). Finally, the subgroup “Early-decliners” was identified in two of the three trials (5% HPTN035, 3% MDP301), characterised by women who initially had a high probability of reporting gel use, but the probability of reporting gel use at last sex act decreased sharply after the first or second visit and remained lower than any of the other trajectories for the remainder of follow-up.

### Factors associated with adherence patterns

The “Consistently-high” subgroup was used as the reference trajectory in the multinomial regression analysis for each trial (Tables 2–4).

**Table 2:** HPTN 035 multivariable multinomial logistic regression.

HPTN 035 multivariable multinomial logistic regression				
	Reference Class: Reported consistently high adherence			
	Early decliners	Variable	Later decliners	
n = 2282	Adjusted RRR	Adjusted RRR	Adjusted RRR	Likelihood ratio chi square test
	[95% CI]	[95% CI]	[95% CI]	
**Age**	2.31	1.62	1.64	p<0.001
Under 30 vs. 30+	[1.34–4.00]	[1.21–2.17]	[1.25–2.15]	
**Site (vs. Durban)**				p<0.001
Philadelphia	2.15	0.96	1.01	
	[0.98–4.71]	[0.55–1.68]	[0.61–1.67]	
Harare	0.39	0.40	0.74	
	[0.13–1.20]	[0.22–0.74]	[0.47–1.16]	
Chitungwiza	0.24	0.24	0.55	
	[0.07–0.82]	[0.12–0.47]	[0.35–0.87]	
Hlabisa	0.83	0.94	0.72	
	[0.39–1.73]	[0.62–1.42]	[0.48–1.09]	
Blantyre	0.62	0.85	0.65	
	[0.30–1.29]	[0.58–1.26]	[0.44–0.95]	
Lilongwe	1.46	1.51	1.22	
	[0.80–2.64]	[1.06–2.14]	[0.87–1.70]	
Lusaka	0.66	0.63	0.80	
	[0.30–1.44]	[0.39–1.00]	[0.54–1.19]	
**Partner dislike of gel**	5.53	3.37	2.57	p<0.001
He did not like it vs. other responses	[2.23–13.75]	[1.67–6.79]	[1.25–5.32]	
**Education**	0.87	1.17	0.95	p = 0.711
Primary or less vs. secondary+	[0.47–1.59]	[0.83–1.64]	[0.69–1.30]	
**Number of sex partners at baseline**	1.68	1.51	1.47	p = 0.503
2–6 vs. 1 in last 3 months	[0.60–4.71]	[0.72–3.13]	[0.75–2.87]	
**Number of vaginal sex acts at baseline**	1.43	1.29	0.96	p = 0.308
5–21 vs. 0–4 in past week	[0.81–2.54]	[0.92–1.82]	[0.70–1.33]	
**Condom use at baseline**	0.86	0.88	0.81	p = 0.361
No vs. yes to condom at last sex act	[0.55–1.34]	[0.68–1.14]	[0.64–1.04]	
**Anal sex at baseline**	2.52	1.36	1.82	p = 0.103
Yes to anal sex ever vs. no	[1.02–6.21]	[0.67–2.73]	[0.99–3.34]	
**Exchanged sex for money, etc. at baseline**	0.31	0.44	0.73	p = 0.305
Yes vs. no	[0.03–3.04]	[0.14–1.38]	[0.32–1.66]	
**Participant thought gel was messy**	1.07	1.17	1.19	p = 0.857
Yes vs. other responses	[0.50–2.32]	[0.72–1.89]	[0.76–1.85]	
**Participant thought the gel was difficult to remember**	1.26	0.90	0.83	p = 0.694
Yes vs. other responses	[0.64–2.49]	[0.57–1.42]	[0.53–1.30]	
**Participant liked the gel because they thought it may protect against HIV**	1.32	0.96	1.05	p = 0.672
No vs. yes	[0.82–2.14]	[0.71–1.30]	[0.80–1.37]	
**Participant reported that their partner liked the gel the last time they used it**	1.10	1.14	1.18	p = 0.555
Other responses vs. yes he liked it	[0.68–1.79]	[0.87–1.50]	[0.92–1.51]	

Variables at the top of the table show an association with latent adherence trajectory at the p = 0.05 level based on the likelihood ratio chi-square-ratio test and are adjusted for each other. Variables at the bottom of the table did not meet the criterion for being included in the model as having been associated with the latent adherence trajectories, but are included so that they may be compared across the trials. Variables in this section are adjusted for the variables which were associated with latent adherence trajectory, but not adjusted for the other variables which did not meet the criterion of p = 0.05 for the likelihood ratio chi-square test.

**Table 3:** MDP 301 multivariable multinomial logistic regression.

MDP 301 multivariable multinomial logistic regression				
	Reference Class: Reported consistently high adherence			
	Early decliners	Variable	Later decliners	
n = 5083	Adjusted RRR	Adjusted RRR	Adjusted RRR	Likelihood ratio chi square test
	[95% CI]	[95% CI]	[95% CI]	
**Age**	1.80	1.21	1.53	p<0.001
Under 30 vs.30+	[1.25–2.60]	[1.04–1.42]	[1.24–1.88]	
**Site vs. Durban**				p<0.001
Joburg	2.24	1.77	1.25	
	[1.50–3.33]	[1.47–2.14]	[0.98–1.60]	
Masaka	0.49	1.05	0.62	
	[0.19–1.28]	[0.77–1.43]	[0.39–0.99]	
Mwanza	1.00	0.92	0.96	
	[0.52–1.91]	[0.69–1.23]	[0.67–1.37]	
Africa Centre	0.77	0.72	0.79	
	[0.38–1.57]	[0.53–0.97]	[0.54–1.15]	
Mazabuka	0.46	0.88	0.91	
	[0.22–0.95]	[0.68–1.13]	[0.66–1.25]	
**Number of sex acts at baseline**	1.28	1.27	1.16	p = 0.017
4+ or more last week vs. 3 or less	[0.91–1.81]	[1.08–1.48]	[0.94–1.44]	
**Education**	0.79	0.76	0.79	p = 0.009
Primary or less vs. secondary +	[0.55–1.14]	[0.64–0.91]	[0.63–1.00]	
**Number of sex partners at baseline**	1.67	1.19	1.62	p = 0.757
>1 partner in last week vs. 1	[0.35–7.98]	[0.51–2.78]	0.62–4.25]	
**Condom use at baseline**	0.92	0.88	1.05	p = 0.329
Yes vs. no condom at last sex	[0.66–1.30]	[0.75–1.02]	[0.85–1.29]	
**Anal sex at baseline**	2.21	1.33	0.81	p = 0.397
Yes anal sex in last 4 weeks vs. no	[0.81–6.04]	[0.72–2.45]	[0.30–2.17]	
**Recommend gel to friends if halves women’s risk of HIV?**	0.90	0.86	1.14	p = 0.900
Discourage vs. encourage	[0.27–3.01]	[0.51–1.46]	[0.62–2.10]	
**Was it easy to predict when you needed to use the gel?**	0.57	0.89	0.84	p = 0.167
No vs. yes	[0.34–0.96]	[0.69–1.16]	[0.59–1.18]	
**How likely do you think it is that you might get infected with HIV?**				p = 0.620
Not very likely vs. very likely	0.63	1.00	0.94	
	[0.39–1.02]	[0.80–1.25]	[0.71–1.27]	
Impossible vs. very likely	0.63	0.95	1.04	
	[0.33–1.19]	[0.72–1.24]	[0.72–1.48]	
**Effect of gel on partner’s enjoyment of sex?**	1.08	1.00	0.92	p = 0.990
Less enjoyable vs. other responses	[0.42–2.78]	[0.67–1.48]	[0.53–1.60]	
**Partner dislike of gel**	2.01	1.50	1.26	p = 0.165
Disliked it vs. other responses	[0.87–4.64]	[0.98–2.29]	[0.68–2.33]	

Variables at the top of the table show an association with latent adherence trajectory at the p = 0.05 level based on the likelihood ratio chi-square-ratio test and are adjusted for each other. Variables at the bottom of the table did not meet the criterion for being included in the model as having been associated with the latent adherence trajectories, but are included so that they may be compared across the trials. Variables in this section are adjusted for the variables which were associated with latent adherence trajectory, but not adjusted for the other variables which did not meet the criterion of p = 0.05 for the likelihood ratio chi-square test.

**Table 4:** Carraguard multivariable multinomial logistic regression.

Carraguard multivariable multinomial logistic regression			
	Reference Class: Reported consistently high adherence		
	Later decliners	Variable	
n = 6039	Adjusted RRR	Adjusted RRR	Likelihood ratio chi square test
	[95% CI]	[95% CI]	
**Age**	1.63	1.40	p < .001
Under 35 vs. 35+	[1.26–2.11]	[1.06–1.86]	
**Site**			p < .001
Medunsa (vs. UCT)	0.48	0.39	
	[0.34–0.66]	[0.27–0.55]	
MRC (vs. UCT)	1.94	1.24	
	[1.49–2.52]	[0.92–1.66]	
**Number of sex partners at baseline**	1.56	1.74	p = 0.008
2 or more vs. 1	[1.06–2.29]	[1.13–2.67]	
**What was your most recent partner’s reaction to you using the study gel?** *	3.26	3.98	p = 0.016
Refused me to use gel vs. other responses	[1.22–8.69]	[1.30–12.17]	
**Education** *	0.79	0.82	p = 0.621
Primary or less vs. secondary +	[0.44–1.40]	[0.42–1.60]	
**Number of sex acts baseline**	1.08	1.11	p = 0.677
5 or more sex acts in past 2 weeks vs. 4 or less	[0.83–1.41]	[0.83–1.48]	
**Condom use at baseline**	0.99	0.91	p = 0.882
No condom used at last sex act with steady partner vs. yes condom	[0.71–1.39]	[0.62–1.33]	
**Anal sex at baseline**	0.94	1.24	p = 0.844
Yes unprotected anal sex in the past 3 months vs. no	[0.47–1.89]	[0.58–2.62]	
**Have you ever had sex in exchange for money?**	0.99	0.72	p = 0.773
yes vs no	[0.48–2.06]	[0.27–1.86]	
**What effect did the study gel have on your sexual pleasure?** *			p = 0.531
Less pleasure vs. more pleasure	2.20	0.44	
	[0.77–6.29]	[0.04–4.91]	
No effect vs. more pleasure	1.09	1.18	
	0.67–1.79	[0.67–2.09]	
**What effect did the gel have on your most recent partner’s sexual pleasure?** *	1.38	0.99	p = 0.859
Less pleasure vs. other responses	[0.46–4.10]	[0.24–4.09]	

*Subsample of 1191 participants.

Variables at the top of the table show an association with latent adherence trajectory at the p = 0.05 level based on the likelihood ratio chi-square-ratio test and are adjusted for each other. Variables at the bottom of the table did not meet the criterion for being included in the model as having been associated with the latent adherence trajectories, but are included so that they may be compared across the trials. Variables in this section are adjusted for the variables which were associated with latent adherence trajectory, but not adjusted for the other variables which did not meet the criterion of p = 0.05 for the likelihood ratio chi-square test.

#### Age

Across all trials (Tables 2–4) older age, versus younger age, was associated with belonging to the latent adherence trajectories which consistently reported gel use at last sex act (“consistently-high” group). In all trials, younger women had a greater chance than older women of belonging to an adherence trajectory that reported less consistent adherence to using the study gel at last sex act: they were more likely to be a part of the “Later-decliners”, “Variable-adherers” or “Early-decliners” trajectories, compared to the consistently-high adherence-reporting group.

#### Site

In this study, model results consistently showed strong evidence across all trials that site was associated with latent adherence trajectory (Tables 2–4). The direction of the association depended on which sites were being compared.

#### Reported negative reaction of partner to gel

In the adjusted analysis, there was strong evidence that reported partner-dislike or refusal of gel was associated with an increased chance of being in an adherence trajectory that did not consistently report gel use in two of the three trials included in this study (HPTN035, Table 2; Carraguard, Table 4). For example, in HPTN035, women who reported that their partner disliked the gel, compared to those who didn’t, were over 5 times more likely to be in the early-decliners group than in the consistently-high group (aRRR = 5.53, CI = 2.23–13.75), and around 3 times more likely to be in the later-decliners or variable-adherence groups, compared with women who did not report partner-dislike of gel. Similarly, in Carraguard, women who reported that their partner refused them to use gel were around 3 times more likely to be in the later-declining group compared to the consistently-high adhering group, and around 4 times more likely to be in the variable-adherence group, compared to women who provided other responses. In MDP301, in the age-adjusted analysis, reported partner-dislike of gel was associated with an increased chance of belonging to an adherence trajectory that did not consistently report gel use; however this association was not evident in the fully-adjusted analysis.

#### Number of sex partners reported at baseline

In the Carraguard trial, there was strong evidence that reported higher number of recent sex partners at baseline was associated with belonging to latent trajectories that did not consistently report gel use (Table 4). Women who had 2 or more partners were nearly 2 times more likely to be in the later-decliner or variable-adherence groups than in the consistently-high group (aRRR = 1.56, CI = 1.06–2.29 and aRRR = 1.74, CI = 1.13–3.67), compared to women who reported only 1 partner at baseline. In the other trials, although women with a higher number of partners at baseline were more likely to belong to an adherence group that did not consistently report gel use, the association was not statistically significant.

#### Number of sex acts reported at baseline

In the MDP301 trial, women who reported a higher number of recent sex acts at baseline had a higher chance of belonging to an adherence trajectory that did not consistently report gel use (Table 3), compared to women who reported fewer sex acts. Women who had ≥4 sex acts were about 1.3 times more likely to be in the early-decliner or variable-adherence groups than in the consistently high group, and about 1.2 times more likely to be in the late-decliner group, compared with women who reported 3 or less sex acts. In other trials, there was a trend for women who reported a higher number of sex acts to have an increased chance of being in an adherence trajectory which did not consistently report using gel: however, effect sizes were generally small, and the association was not statistically significant (Tables 2 & 4).

#### Education

In MDP301, there was strong evidence that women with less education had an increased chance of membership of the adherence trajectory that consistently reported gel use at last sex act. (Table 3).

Women with only primary education or less were around 20% less likely to be in the early-decliner, variable, or late-decliner adherence groups, than in the consistently-high adherence group, compared with women with secondary education or higher.

A similar trend of less education being associated with an increased chance of belonging to the adherence group which reported consistent gel use was seen in the Caraguard trial and less so in HPTN035 (Tables 2 & 4).

## Discussion

### Patterns of adherence

Latent class analysis identified subgroups of participants with different adherence patterns in all three trials in this exploratory study, using self-reported data. Latent adherence trajectories across the trials were similar. Common to all three trials were larger subgroups of participants who reported “Consistently-high” adherence, a subgroup of “Later-decliners” and a subgroup of “Variable-adherers.” A small subgroup characterised as “Early-decliners” was identified in two of the three trials.

### Factors associated with adherence patterns

There was strong evidence that site was associated with latent adherence trajectory membership in all three of the included trials. This association has been observed in other studies from the included trials [20–22] and other HIV prevention trials such as MIRA [23], and FEM-PrEP [24]. This finding indicates that local culture and site staff factors may have played a role in how participants reported their adherence or may have influenced participants’ actual adherence. In some locations, underlying culture may influence participants to report higher adherence to “please” site staff, or participants may report gel use in order to avoid being reprimanded, removed from the trial, or showing vulnerability [25–30].

Younger age, in all three trials, was found to be associated with the subgroups of participants that did not consistently report high adherence; this finding has been observed in other HIV prevention studies [3, 21–23, 31–33]. Reasons that might affect younger women’s ability to consistently use the gel could be that they are less likely to be in stable relationships, that they have more partners, and that they may be less able to plan sex. Older women may be more aware of their HIV risk, recognising non-faithful partners. Older women may be more likely to be mothers, and more interested in protecting themselves and their children from HIV. They may be living in more stable households where logistically they can better manage gel use, compared to younger participants who may be having sex outside of locations where they reside [3]. Older participants, compared to younger participants, may also have more self-efficacy and be better prepared to negotiate gel use with their partners or decide to use it even without partner approval.

It is not surprising that if a participant reports her partner did not like the gel or refused her use of the gel, her adherence to gel would be negatively affected. While these data were collected at study exit, after participants reported their adherence, these results are consistent with the growing body of evidence showing partner dynamics are a critical factor affecting female participants’ adherence in biomedical HIV prevention trials as well as affecting women’s concerns about PrEP use [22, 25, 28, 29, 34–43].

The impetus for vaginal microbicide development came from the reality that many male partners refuse to use condoms and women need an HIV prevention strategy they can control, and yet this same dynamic continues to be a barrier for female participants to use the very product being tested to free themselves from that constraint. This speaks to the real and ongoing challenges that women face in sexual relationships with men. HIV prevention trial teams, with the consent of female participants, can design more comprehensive and systematic opportunities to engage male partners at the beginning of trials and throughout follow-up [22, 34, 39]. Clinical trials and programmes rolling-out proven HIV prevention methods will benefit from greater understanding of these dynamics, providing more support to women to effectively communicate with their male partners, ultimately empowering women to use HIV prevention products more effectively.

Women who reported a higher number of sex acts at baseline in MDP301 and a higher number of sex partners at baseline in Carraguard were associated with membership of the latent trajectories that did not consistently report high adherence. These results are in agreement with trial teams’ own findings in other analyses that women at greater risk of HIV have less consistent adherence [20, 21]. Those who report more sex and sex partners at baseline might be exchanging sex, living or working in less stable environments, and be more mobile, all of which may make it challenging to use gel at each sex act. Participants with fewer sex partners and sex acts at baseline might represent a population of women in more stable relationships, with more established home situations, including locations for gel storage.

There was strong evidence that less education was associated with membership of the trajectory that consistently reported high adherence in MDP301. This was in agreement with MDP301’s own findings when looking at predictors of consistent adherence [21]. Results from the other trials, however, were variable. There is not clear evidence on how education may be related to reported adherence. Possibly women with less education have less economic stability, may fear removal from the trial and loss of reimbursements, and thus might be more inclined to report “good” adherence. Also possible is that participants with more education feel freer to not follow directions from clinic staff or freer to report their actual adherence compared to women with less education.

### Latent class analysis as a tool for understanding adherence to HIV prevention products

In this study, the use of LCA was effective at identifying different patterns of adherence among a seemingly homogenous population of microbicide gel trial participants. This information was obtained with self-reported data, which is less expensive than biomarker data, and preserves clinical trial blinding. Identifying adherence trajectories and associated factors can help trial teams better understand their participant populations and better address suboptimal adherence issues.

For example, early-decliners may represent a proportion of women who joined the trial without the intention of using the study gel. Alternatively, they may have found using the gel initially difficult and may have benefited from targeted support on product use. Later-decliners may represent participants who stop using the gel for several reasons–such as real-life barriers that make gel use around the time of sex difficult and may represent women who would have benefitted from more intensive support around how to communicate with their male partners about the gel. Low reported adherence at one site might indicate significant barriers to gel use, and the need for understanding and engagement around cultural barriers to adherence. Very high reported adherence at one site might indicate better adherence, or could indicate that staff have poor rapport with participants, such that participants are not comfortable being honest. Trial teams can use such information to investigate circumstances, liaise with stakeholders to understand their needs, and re-train staff where necessary. Information learned through follow-up can be integrated into trial procedures prospectively during implementation or for future studies to better support participant recruitment, product adherence, adherence reporting, and trial conduct, as well as planning and rollout of programmes for approved biomedical HIV prevention methods.

### Limitations

There are several limitations of this study. All adherence and behavioural data are self-reported. Data may be biased towards high adherence as participants may have been motivated to please staff members or feared removal from the trial [25–27, 29, 30, 40, 44]. The Carraguard trial asked only one question about gel use (the primary adherence measure was not self-report), which may have biased results towards higher self-reported adherence. Certain sexual behaviours may be underreported for fear of judgement by staff [45, 46]. Participants reported their perceived partner views of gels at study exit, after reporting their own adherence. Theoretically, women could have decided to state that their partners had a negative view of the gels as an “excuse” for their own non-adherence. If that occurred to a large extent, that could produce an association between low adherence and partner dislike of the gels that was stronger than in reality.

In this analysis, factors such as number of sex partners, condom use, and transactional sex at baseline were examined in relation to latent adherence trajectories. In reality, factors such as these may have changed over the course of trial participation. The multivariable models were built using a p-value threshold of 0.05 for the likelihood ratio chi-square test. This method has a high risk for type II error, particularly if a candidate factor is confounded in an equal and opposite direction by another factor not in the model.

The data included in this analysis were collected from trials that ended most recently in 2009. Thus, these data are from a period of time prior to antiretroviral microbicides. Since these trials were conducted, the field of biomedical HIV prevention has moved on to antiretrovirals and products that can be used for longer periods of time, such as vaginal rings and injectable pre-exposure prophylaxis (PrEP).

In this analysis, LCA was used to better understand longitudinal reporting patterns of gel use among women in the included microbicide trials. In LCA, there is no accepted best method for selecting the number of latent classes in a particular analysis, therefore different investigators might come to different conclusions based on their evaluations of criteria and usefulness of the various models. Once a model has been selected with a specified number of latent classes, the model will mathematically separate the population into the specified number of trajectories. LCA is a model which simplifies large amounts of data. In reality, each participant has her own trajectory of gel use over time. While the analysis is a simplification of reality, this simplification has supported interpretation of adherence in these trial populations.

## Conclusion

LCA combined with multinomial logistic regression is a method HIV prevention trials and programmes rolling out proven HIV prevention methods can use to interpret adherence data from their populations. This information can support identification of issues related to product use. Trial teams and programme implementers can then engage with communities to better understand barriers to adherence and develop strategies together to support optimal use of these methods [47]. An important factor associated with lower adherence in two trials in this study was perceived negative reaction of male partners to the study gel. These findings are consistent with results from other clinical trials and programmatic rollout of biomedical HIV prevention methods which highlight concerns expressed by women in Africa about the impact of relationship dynamics when considering or using HIV prevention methods. This study contributes to the body of evidence that women need more support to navigate power dynamics within their relationships with male partners so that they can successfully use HIV prevention methods.

## Supporting information

S1 Appendix Latent adherence trajectory information and model selection for 3 trials. (DOCX)
