# Supplementary material for: Hidden heterogeneity: Uncovering patterns of adherence in microbicide trials for HIV prevention
Source: PLoS One. 2022 May 12;17(5):e0267011. doi: 10.1371/journal.pone.0267011 (PMC9098085; doi:10.1371/journal.pone.0267011)
Supplement: S1 Appendix — (DOCX) [file pone.0267011.s001.docx]

**SI Appendix**

**Latent adherence trajectory information and model selection for 3 trials**


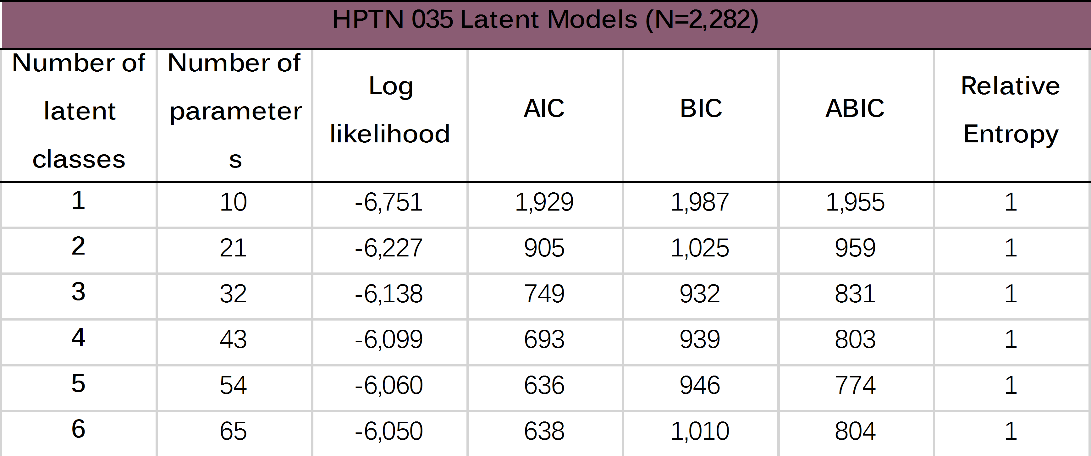


**Table S1. HPTN 035 latent trajectory information**

Table S1 provides information about each of the models for HPTN 035 with 1–6 latent

adherence trajectories. The 5-class model had the lowest AIC and ABIC indices of the six models (636 and 774, respectively). The 3-class model had the lowest BIC value of the six models (932). Thus, model selection was among models with 3, 4, or 5 latent classes. Considering the fit statistics along with parsimony and interpretability, the model with 4 classes was selected as it provided enough information about the population and was easily interpretable, unlike the 5-class model.


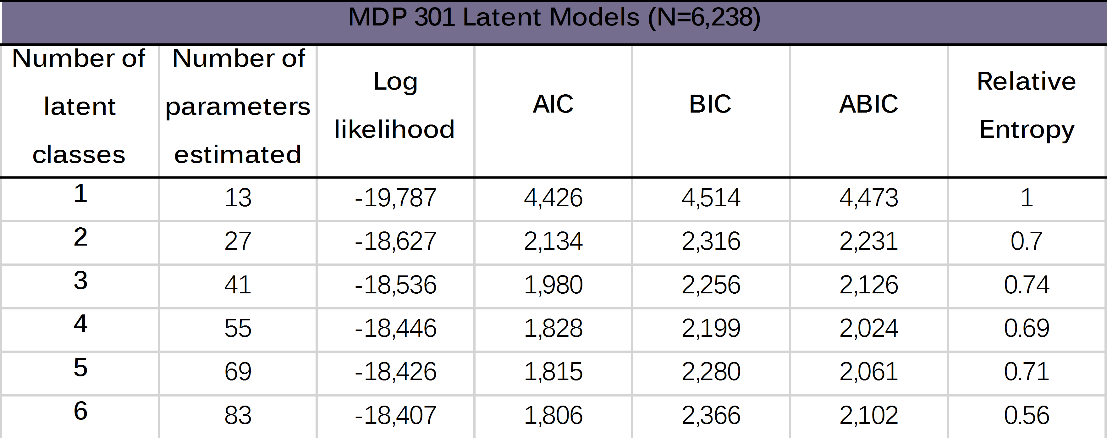


**Table S2. MDP 301 latent trajectory information**

Table S2 provides information about each of the models for MDP 301 with 1–6 latent

adherence trajectories. The 6-class model had the lowest AIC value (1806) whereas the 4-class model had the lowest values for both the BIC and ABIC (2199 and 2024, respectively). As the 4-class model also fit the criteria for parsimony and interpretability, the 4-class model was selected.


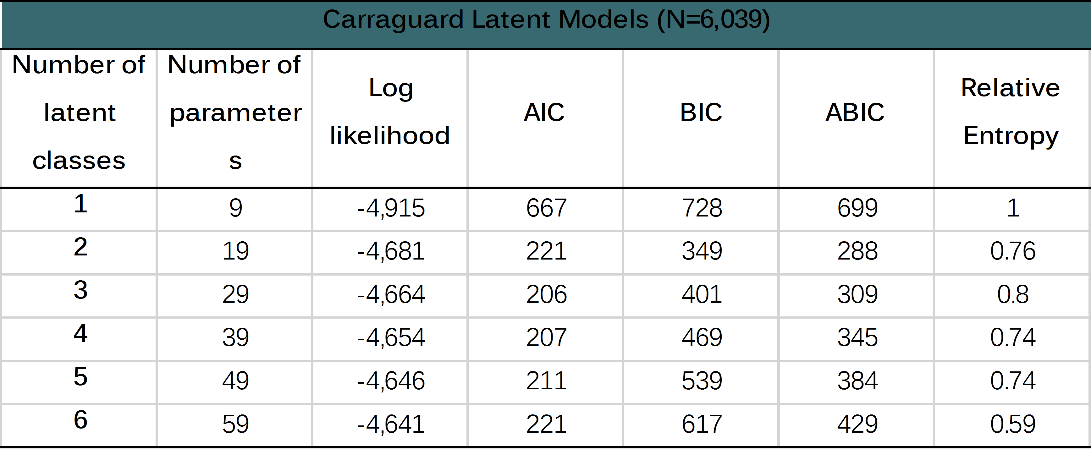


**Table S3. Carraguard latent trajectory information**

Table S3 provides information about each of the models for Carraguard with 1–6 latent

adherence trajectories. The 3-class model had the lowest AIC index (206), whereas the 2-class model had the lowest BIC and ABIC indices (349 and 288, respectively). Thus, selection was between the 2-class and 3-class models. As the 3-class model was easily interpretable, provided more information than the 2-class model, and had large enough proportions of the population belonging to each class, the 3-class model was selected.
